# Optimal Timing of Cholecystectomy for Patients with Concurrent Acute Cholecystitis and Acute Cholangitis after Successful Biliary Drainage by Interventional Endoscopic Retrograde Cholangiopancreatography

**DOI:** 10.3390/jcm11216603

**Published:** 2022-11-07

**Authors:** Yau-Ren Chang, Chi-Huan Wu, Huan-Wu Chen, Yu-Liang Hung, Chia-Hsiang Hu, Ruo-Yi Huang, Min-Jung Wu, Hao-Wei Kou, Ming-Yang Chen, Chun-Yi Tsai, Shang-Yu Wang, Keng-Hao Liu, Jun-Te Hsu, Chun-Nan Yeh, Nai-Jen Liu, Yi-Yin Jan

**Affiliations:** 1 Division of General Surgery, Linkou Chang Gung Memorial Hospital, Taoyuan 333, Taiwan; 2 Department of Gastroenterology and Hepatology, Linkou Chang Gung Memorial Hospital, Taoyuan 333, Taiwan; 3 Division of Emergency and Critical Care Radiology, Department of Medical Imaging and Intervention, Linkou Chang Gung Memorial Hospital, Taoyuan 333, Taiwan; 4 Department of General Surgery, Jen Ai Chang Gung Health, Dali Branch, Taichung 412224, Taiwan; 5 School of Traditional Chinese Medicine, Chang Gung University, Taoyuan 333, Taiwan

**Keywords:** cholecystectomy, cholecystitis, cholangitis, endoscopic retrograde cholangiopancreatography, ERCP, laparoscopic cholecystectomy, cholecystolithiasis, cholelithiasis, gallbladder stones

## Abstract

*Background*: Concurrent acute cholecystitis and acute cholangitis is a unique clinical situation. We tried to investigate the optimal timing of cholecystectomy after adequate biliary drainage under this condition. *Methods*: From January 2012 to November 2017, we retrospectively screened all in-hospitalized patients undergoing endoscopic retrograde cholangiopancreatography (ERCP) and then identified patients with concurrent acute cholecystitis and acute cholangitis from the cohort. The selected patients were stratified into two groups: one-stage intervention (OSI) group (intended laparoscopic cholecystectomy at the same hospitalization) vs. two-stage intervention (TSI) group (interval intended laparoscopic cholecystectomy). Interrogated outcomes included recurrent biliary events, length of hospitalization, and surgical outcomes. *Results*: There were 147 patients ultimately enrolled for analysis (OSI vs. TSI, 96 vs. 51). Regarding surgical outcomes, there was no significant difference between the OSI group and TSI group, including intraoperative blood transfusion (1.0% vs. 2.0%, *p* = 1.000), conversion to open procedure (3.1% vs. 7.8%, *p* = 0.236), postoperative complication (6.3% vs. 11.8%, *p* = 0.342), operation time (118.0 min vs. 125.8 min, *p* = 0.869), and postoperative days until discharge (3.37 days vs. 4.02 days, *p* = 0.643). In the RBE analysis, the OSI group presented a significantly lower incidence of overall RBE (5.2% vs. 41.2%, *p* < 0.001) than the TSI group. *Conclusions*: Patients with an initial diagnosis of concurrent acute cholecystitis and cholangitis undergoing cholecystectomy after ERCP drainage during the same hospitalization period may receive some benefit in terms of clinical outcomes.

## 1. Introduction

Acute cholecystitis (AC) and acute cholangitis (ACL) are common biliary diseases in daily practice. Both AC and ACL are regarded as complicated symptomatic cholecystolithiasis. ACL is a sequential condition due to obstruction of the bile duct, mostly due to biliary stones traveling down from the gallbladder. The demographic data, pathophysiology, and treatment of these patients have been extensively studied, and several clinical guidelines, such as the 2018 Tokyo Guidelines (TG18) and World Society of Emergency Surgery (WSES) guidelines, have proposed integrated principles of management for both AC and ACL [1,2,3]. Published studies have shown that in Western populations, approximately 10% to 20% of patients who undergo cholecystectomy due to cholecystolithiasis have coexisting choledocholithiasis [4], with an even higher percentage, up to 30%, in the Chinese population. In addition to coexisting cholecystolithiasis and choledocholithiasis, concurrent AC and ACL is another unique variant encountered during daily clinical practice, and correctly diagnosing the coexistence of both diseases is not easy because of overlapping clinical presentations in some aspects. While the clinical guidelines have well addressed the management of AC and ACL, management guidelines for AC complicated with ACL have not been proposed. Owing to the lack of diagnostic principles and poor definition of this specific group, there have been no convincing data to indicate the actual incidence and guide proper management of this complicated biliary condition. Although cholecystectomy is the definitive treatment for symptomatic cholecystolithiasis, biliary drainage, especially endoscopic retrograde cholangiopancreatography (ERCP), is an important modality for impacted bile duct stones, ACL, and biliary pancreatitis. For impacted common bile duct stones, cholecystectomy has been proposed within two weeks after ERCP [5]. However, there is no evidence for the standard management of cholecystectomy and ERCP planning for patients with concurrent AC and ACL. In the present study, we identified a group of patients with concurrent ACs and ACLs based on current clinical guidelines [1,2], and all patients had undergone successful biliary drainage via ERCP. We tried to investigate the optimal timing of cholecystectomy after biliary drainage in this selected cohort of concurrent AC and ACL.

## 2. Methods and Materials

**Figure 1:**
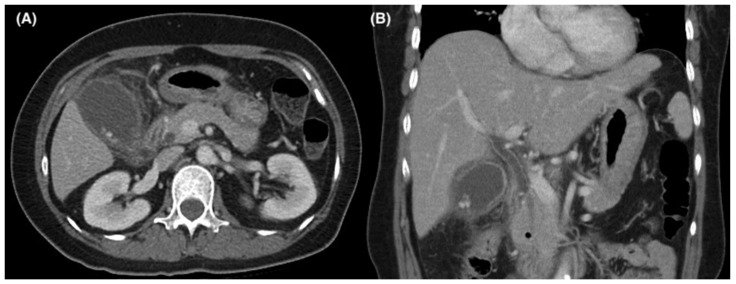
Axial (**A**) and coronal (**B**) computed tomography images of a patient with concurrent acute cholecystitis and cholangitis.

From January 2012 to November 2017, we retrospectively screened all in-hospitalized patients undergoing ERCP at Chang Gung Memorial Hospital (CGMH). This study was approved by the Internal Review Board of CGMH under reference number 201801210B0. Due to the retrospective design of our study, informed consent was waived by the ethics committee for the entire study. We then identified patients with concurrent AC and ACL from the aforementioned cohort. The group of interest patients was clinically diagnosed with concurrent AC and ACL (cACC). The diagnostic criteria of cACC in the present study included (1) clinical symptoms/signs such as tenderness over the right upper abdomen, Murphy’s sign, and fever > 38 °C; (2) laboratory findings such as white blood cell count <4000 or >10,000/μL, CRP ≥ 1 mg/dL and total bilirubin ≥ 2 mg/dL; (3) evidence of biliary dilatation, etiology of biliary stone/stricture and findings characteristic of AC on imaging studies (Figure 1); and (4) pus-like or turbid drainage content observed under endoscopic view when ERCP was conducted. A diagnosis of biliary pancreatitis was also made, defined as (1) epigastric pain, especially radiating to the back; (2) initial serum lipase level and amylase level at least three times greater than upper limits of normal range; and (3) characteristic findings of acute pancreatitis on CT imaging. Biliary pancreatitis was identified before therapeutic management strategies, such as ERCP, antibiotics, or surgical intervention, were applied. Patients who underwent ERCP after cholecystectomy and those with dysfunction of at least one organ, a biliary or hepatic malignancy, a history of previous biliary tract infection or abdominal surgery, or Mirrizi’s syndrome were all excluded. Since intended laparoscopic cholecystectomy (LC) is now the standard for symptomatic cholecystolithiasis, we also excluded patients who underwent intended open cholecystectomy.

We then categorized the selected patients into a “one-stage intervention” group (OSI group), which received successful ERCP and urgent cholecystectomy at the same hospitalization, and a “two-stage intervention” group (TSI group), which received successful ERCP for ACL and conservative treatment for AC with interval cholecystectomy later.

### 2.1. Therapeutic Strategies for Patients with Concurrent Acute Cholangitis and Acute Cholecystitis

Empiric antibiotics with adequate hydration were prescribed as initial treatment. The antibiotic regimen may be adjusted later based on the results of microbiology tests or unsatisfying clinical responses regarding initial empiric drugs. Every patient underwent ERCP with endoscopic papillotomy for successful biliary decompression and/or stone retrieval. Once cholangitis had improved, cholecystectomy, percutaneous transhepatic gallbladder drainage (PTGBD), or conservative treatment with antibiotics only was then arranged.

### 2.2. Preoperative Assessment and Surgical Procedures of Cholecystectomy

After the cACC patients in our cohort underwent biliary drainage, cholecystectomy was arranged during the same hospitalization or performed later (interval cholecystectomy). Preoperative evaluations included plain chest film, electrical cardiography, laboratory tests, information on underlying conditions, and anesthetic risks. Intended LC was arranged for each patient using a standardized four-port or modified three-port procedure in selected patients. Conversion to the open procedure, namely, laparotomy, was indicated based on the surgeons’ judgment.

### 2.3. Clinical Information

Clinical data, including sex, age, Charlson Comorbidity Index (CCI), American Society of Anesthesiology (ASA) score, clinical presentation, duration of the course, and disease severity, were all extracted from the medical records. Laboratory data were also collected, including complete blood count, C-reactive protein, bilirubin, hepatic enzymes, amylase, lipase, creatinine, and coagulation factors. The results of microbiological examination of pathogens were also obtained. Imaging studies, such as abdominal sonography, abdominal computed tomography, ERCP, and magnetic resonance cholangiopancreatography, were reviewed to confirm the diagnosis of the enrolled subjects.

### 2.4. Outcome Evaluation

General outcomes included recurrent biliary events (RBE), length of stay of first hospitalization (1st LOS), and total length of hospitalization (tLOS). An RBE was defined as recurrent cholecystitis or recurrent cholangitis following the first admission. tLOS is the total number of hospitalization days and includes first admission, admission for interval LC, and admission for RBE during the follow-up period. We also assessed the surgical outcome of these two groups in our cohort. The surgical outcomes consisted of the operative time of cholecystectomy, postoperative days until discharge, intraoperative blood transfusion, conversion to open procedure, and postoperative complications, including superficial site infection, postoperative bile leakage, intra-abdominal abscess, postoperative sepsis, and intra-abdominal bleeding.

### 2.5. Recurrent Biliary Events

For patients in the OSI group, RBE or overall RBE was defined as the first identified RBE during the follow-up period. For patients in the TSI group, two different RBEs were determined based on the time point of surgery: overall RBE and RBE after interval surgery. Since RBE is one of our outcomes of interest, we compared the OSI group with the TSI group in terms of (1) overall RBE vs. overall RBE and (2) overall RBE vs. RBE after interval surgery. Independent risk factors for RBE were also analyzed.

### 2.6. Statistical Analysis

We utilized R statistics and Statistical Package for Social Sciences (SPSS) for the analyses. Statistical methods included the chi-square test, Fisher’s exact test for categorical variables, and Mann–Whitney U-test for continuous variables. Logistic regression was used to predict risk factors for associated data. Values of *p* < 0.05 were considered statistically significant.

## 3. Results

**Figure 2:**
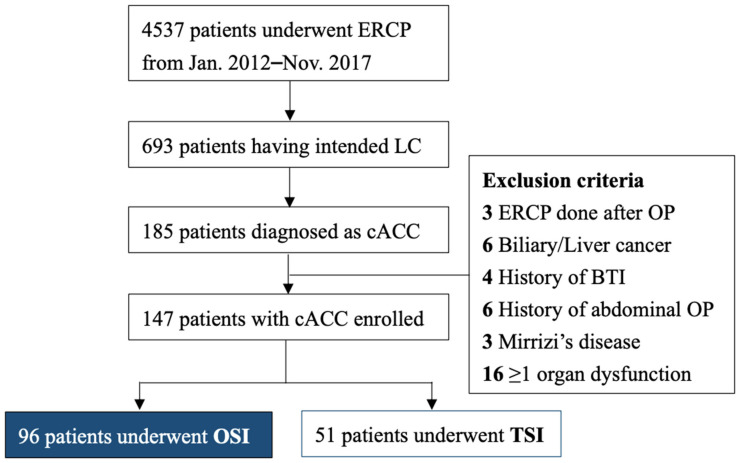
Inclusion and exclusion criteria for the patients diagnosed with concurrent acute cholecystitis and acute cholangitis and later underwent intended laparoscopic cholecystectomy. ERCP, endoscopic retrograde cholangiopancreatography; LC, laparoscopic cholecystectomy; OP, operation; cACC, concurrent acute cholecystitis and cholangitis.

From January 2012 to November 2017 (Figure 2), 4537 patients diagnosed with cholelithiasis and biliary obstruction underwent ERCP in our institute, of whom 693 underwent intended LC. Among this subpopulation, 185 were diagnosed with cACC. After excluding 3 patients with ERCP performed after cholecystectomy, 16 patients with dysfunction in at least one organ, 6 patients with biliary or hepatic malignancy, 4 patients with a history of biliary tract infection, 6 patients with a history of abdominal surgery, and 3 patients with Mirrizi syndrome, 147 patients were ultimately enrolled in our study. Ninety-six of them underwent OSI, and fifty-one underwent TSI. There was no significant difference in the clinical data between the two groups (Table 1). In the TSI group, the median duration from ERCP to definitive cholecystectomy was 2.5 months.

### 3.1. Analysis of General Outcomes

In the general outcome analysis (Table 1), the 1st LOS was significantly longer in the OSI group than in the TSI group (8.50 days vs. 7.18 days, *p =* 0.001). In contrast to the 1st LOS, the tLOS was significantly shorter (9.00 days vs. 17.87 days*, p < 0*.001) in the OSI group than in the TSI group. There was no in-hospital mortality in both groups. The causes of five cases of overall mortality were aspiration pneumonia (n = 2), acute on chronic renal failure (n = 1), pulmonary embolism (n = 1), and terminal stage of lung adenocarcinoma (n = 1), and there was no significant difference in overall mortality (4.17% vs. 1.96%*, p =* 0.659) between the two groups.

In the RBE analysis, the OSI group presented a significantly lower incidence of overall RBE (5.21% vs. 41.18%, *p* < 0.001) than the TSI group, but no significant difference in RBE was found between the two groups after cholecystectomy done (5.21% vs. 13.73%, *p* = 0.111).

### 3.2. Risk Factors for RBEs

Only TSI was independent risk for RBE in the multivariant analysis (OR = 12.74, 95% CI of OR = 4.42–36.74, *p* < 0.001). Biliary pancreatitis diagnosed before ERCP was noted in 19 patients (12.9%), but it had no significant effect on the surgical outcome or RBEs (OR = 1.17, 95% CI of OR = 0.31–4.34, *p* = 0.816) (Table 2). 

### 3.3. Analysis of Surgical Outcomes

**Figure 3:**
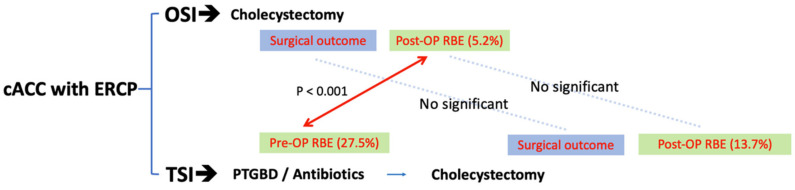
Graphic summary of results of present work. cACC, concurrent acute cholecystitis and cholangitis; OSI, one-stage intervention; TSI, two-stage intervention; RBE, recurrent biliary event; PTGBD, percutaneous transhepatic gallbladder drainage.

Regarding surgical outcomes (Table 3), there was no significant difference between the OSI group and TSI group in general, including in intraoperative blood transfusion (1.04% vs. 1.96%, *p* = 1.000), conversion to open procedure (3.12% vs. 7.84%, *p* = 0.236), postoperative complications (6.25% vs. 11.76%, *p* = 0.342), operation time (118.01 min vs. 125.76 min, *p* = 0.869), and postoperative days until discharge (3.37 days vs. 4.02 days, *p* = 0.643). A graphic summary demonstrates the results of the present work (Figure 3).

At our institution, 1400 to 1500 (2015~2019) patients undergo LC annually, and the average conversion rate is approximately 1.5%, which is lower than that of the cACC group in our study (4.76%).

## 4. Discussion

Most cases of cholelithiasis, or biliary stones, originate from the gallbladder; de novo, biliary stones from the bile duct are relatively rare [6]. Gallbladder stones may elicit symptoms that are localized in the gallbladder, and symptoms resulting from stone migration, such as impacted common bile duct stones and biliary pancreatitis, can also occur. Regarding managing impacted common bile duct stones, ACL, biliary pancreatitis, and acute cholecystitis, cholecystectomy has been recognized as part of the treatment plan. Without cholecystectomy, the greatest concern is recurrent symptoms and biliary infection, a concept similar to RBE in the present study. Lee et al. reported on 100 common bile duct stones patients who underwent ERCP for stone removal. With a mean observation of 18 months, 17% of their cohort suffered from AC, and another 13% was diagnosed with recurrent common bile duct stones [7]. In our previous work on patients with AC undergoing percutaneous cholecystostomy, patients suffered from a cumulative incidence of 29.8% for biliary events within a median follow-up time of 4.27 months [8]. Furthermore, a systematic review of 1841 patients conducted by Loozen et al. showed an overall recurrent gallstone-related disease rate of 22% [9]. Therefore, cholecystectomy seems to be the mainstay of management for complicated cholelithiasis.

The European Society of Gastrointestinal Endoscopy has advised that LC be performed no more than two weeks after successful stone retrieval by ERCP for patients with impacted common bile duct stones, and this practice may optimize clinical outcomes [5]. While simultaneous treatment, namely cholecystectomy, has also been suggested for mild ACL, according to TG 18, half of the experts agreed that the same approach could be used for moderate ACL [1]. In general, cholecystectomy is a definitive treatment for a spectrum of biliary conditions, and the timing of surgery for different situations should be tailored. In the present work, we tried to determine the optimal timing of cholecystectomy under a specific condition of biliary infection, namely concurrent AC and ACL (defined as cACC in the present study). While strategies for managing complicated biliary tract infections or conditions have been proposed in several clinical guidelines or studies with high-level evidence [1,5,10,11], few have addressed the cACC. Therefore, the present study aims to investigate the appropriate strategy for this complicated biliary infection.

Although there is scarce evidence related to the timing of cholecystectomy for cACC, one study from Japan conducted by Abe et al. in 2019 reported on 101 patients diagnosed with both AC and ACL and 151 patients diagnosed with AC only [12]. In their cohort, 78.2% of patients with cACC and 82.1% of AC-only patients underwent LC. While there was no difference in surgical complications, patients with cACC had a longer hospital stay. Abe et al. claimed that early cholecystectomy within 14 days after symptom onset was safely performed for patients with concomitant AC and ACL, showing similar surgical outcomes as patients with AC only. However, patients in their cohort were heterogeneous in several aspects; a significant portion underwent an open procedure, biliary drainage was not performed for every individual due to disease severity, and the rate of conversion to open surgery was not provided. Although relevant evidence is scarce, this challenging clinical situation should be investigated since this can be a scenario encountered in daily practice. In our work, we enrolled patients with cACC only and tried to determine the most suitable strategy: one-stage or two-stage treatment. Our results favored a one-stage intervention strategy.

According to our results, the one-stage intervention strategy, i.e., intended LC after ERCP during the same hospitalization, may confer similar surgical outcomes compared with interval cholecystectomy. In addition, RBE was significantly lower in the OSI group. While the 1st LOS was longer in the OSI group, the tLOS was considerably shorter than the TSI group. In addition, the present study also revealed a similar risk of RBE when patients in the TSI group underwent cholecystectomy. Several studies have focused on the timing of cholecystectomy after successful treatment of ACL without AC [13,14,15]. All these studies demonstrated similar surgical outcomes and RBE compared with our results under cACC conditions. Therefore, regardless of whether AC exists, cholecystectomy should be considered after successful bile duct clearance and drainage. If the patient is physically fit and suffers from a complicated biliary infection, cholecystectomy may be arranged as soon as successful medical treatment and drainage are achieved.

There were several limitations in the present work. First, this was a retrospective study, and individual surgeons made the decision of timing for the intended LC immediately after ERCP. Even without statistical differences in age, ASA, and CCA, we believed that selection bias may still exist. Second, the diagnosis of cACC in our study was simply based on the modification of current criteria for AC and ACL diagnoses. However, the clinical spectrum of AC and ACL may overlap, which is the primary reason why we did not consider the severity of individual conditions (AC and ACL) in our investigation. Third, we did not include time from ERCP to intended LC in the analysis. Since there were few cases in the study, it was difficult to perform further subgroup analysis to precisely define the optimal surgical timing. We can only claim that patients could benefit from surgery in the same hospitalization, but we cannot specifically point out how many days we should wait after ERCP before arranging surgery.

## 5. Conclusions

In conclusion, patients with an initial diagnosis of concurrent AC and ACL, namely cACC in the present study, who are physically capable of tolerating surgery and undergoing ERCP and cholecystectomy during the same hospitalization period may receive some benefit in terms of favorable outcomes. Further studies on a larger scale are necessary to investigate issues related to incidence and severity assessment and to validate the strategy of the one-stage intervention proposed in the present study.

## Figures and Tables

**Table 1:** The analysis of demographic data and recurrent biliary events (RBE) between one-stage intervention (OSI) group and two-stage intervention (TSI) group.

	Total (%/IQR)	OSI (%/IQR)	TSI (%/IQR)	*p*-Value
Sex (n)				0.665
Man	80 (54.42)	51 (53.13)	29 (56.86)	
Woman	67 (45.58)	45 (46.87)	22 (43.14)	
Age ≥ 65 (n)				0.130
Yes	71 (48.30)	42 (43.75)	29 (56.86)	
No	76 (51.70)	54 (56.25)	22 (43.14)	
CCI ≥ 4 (n)				0.276
Yes	3 (2.04)	1 (1.04)	2 (3.92)	
No	144 (97.96)	95 (98.96)	49 (96.08)	
ASA ≥ 3 (n)				0.243
Yes	71 (48.30)	43 (44.79)	28 (54.90)	
No	76 (51.70)	53 (55.21)	23 (45.10)	
Fever ≥ 38*C (n)				0.188
Yes	34 (23.13)	19 (19.79)	15 (29.41)	
No	113 (76.87)	77 (80.21)	36 (70.59)	
RUQ Pain (n)				0.449
Yes	139 (94.56)	92 (95.83)	47 (92.16)	
No	8 (5.44)	4 (4.17)	4 (7.84)	
Duration of Symptoms (day)	2.00 (1.00–5.00)	2.00 (1.00–5.00)	3.00 (2.000–7.000)	0.098
Blood Culture (n)				0.456
Positive	31 (21.09)	22 (22.92)	9 (17.65)	
Negative	116 (78.91)	74 (77.08)	42 (82.35)	
Bile Culture (n)				0.499
Positive	36 (24.66)	22 (22.92)	14 (28.00)	
Negative	110 (75.34)	74 (77.08)	36 (72.00)	
WBC/1000 (/mm^3^)	10.64 (4.544)	9.65 (7.3000–12.700)	9.80 (7.200–15.000)	0.637
CRP (mg/dL)	5.33 (8.137)	13.6 (7.00–49.26)	20.3 (5.88–95.5)	2
Alk-P (U/L)	204.85 (122.487)	178.0 (121.00–255.00)	199.81 (123.824)	0.548
Total Bilirubin (mg/dL)	3.83 (3.492)	3.20 (2.000–5.7000)	3.25 (1.885)	0.226
Biliary Pancreatitis (n)				0.078
Yes	19 (12.93)	9 (9.38)	10 (19.61)	
No	128 (87.07)	87 (90.62)	41 (80.39)	
GB Stone Seen in Pathology (n)				0.507
Yes	125 (85.03)	83 (86.46)	42 (82.35)	
No	22 (14.97)	13 (13.54)	9 (17.65)	
1st LOS (day)	8.60 (4.057)	8.50 (6.25–11.00)	7.18 (3.223)	0.001 *
tLOS (day)	12.71 (9.900)	9.00 (7.00–12.00)	17.87 (14.028)	<0.001 *
In-hospital Mortality (n)				-
Yes	0 (0.0)	0 (0.0)	0 (0.0)	
No	147 (100.0)	96 (100.0)	51 (0.0)	
Overall Mortality (n)				0.659
Yes	5 (3.40)	4 (4.17)	1 (1.96)	
No	142 (96.60)	92 (95.83)	50 (98.04)	
Overall RBE (n)				<0.001 *
Yes	26 (17.69)	5 (5.21)	21 (41.18)	
No	121 (82.31)	91 (94.79)	30 (58.82)	
RBE after Operation (n)				0.111
Yes	12 (8.16)	5 (5.21)	7 (13.73)	
No	135 (91.84)	91 (94.79)	44 (86.27)	

IQR, interquartile range; CCI, Charlson Comorbidity Index; ASA, American Society of Anesthesiologists physical status classification; RUQ, right upper quadrant; Alk-P, alkaline phosphatase; GB, gallbladder; 1st LOS, length of stay of first hospitalization; tLOS, total length of hospitalization. *: *p*-value lower than 0.05 was defined as statistical significance in this study.

**Table 2:** Univariant and multivariant analysis of risk factors for overall recurrent biliary event (RBE).

	OR (%)	95% CI of OR (%)	*p*-Value Univariant	*p*-Value Multivariant
Gender (Women/Men)	3.39	1.29–9.03	0.015 *	0.877
Age ≥ 65 (Yes/No)	1.31	0.56–3.06	0.533	N/S
CCI ≥4 (Yes/No)	2.38	0.21–27.28	0.486	N/S
ASA ≥ 3 (Yes/No)	1.92	0.81–4.57	0.140	N/S
Duration of Symptoms (day)	1.10	1.03–1.19	0.008 *	0.067
WBC/1000 (/mm^3^))	0.91	0.81–1.02	0.096	N/S
Alk-P (U/L)	1.00	0.99–1.01	0.354	N/S
Total Bilirubin (mg/dL)	1.07	0.96–1.19	0.210	N/S
Positive Blood Culture (Yes/No)	1.19	0.40–3.34	0.798	N/S
Positive Bile Culture (Yes/No)	1.04	0.38–2.86	0.933	N/S
Biliary Pancreatitis (Yes/No)	1.17	0.31–4.34	0.816	N/S
TSI (Yes/No)	12.74	4.42–36.74	<0.001 *	<0.001 *

*: *p*-value lower than 0.05 was defined as statistical significance in this study. N/S, not significant. CCI, Charlson Comorbidity Index; ASA, American Society of Anesthesiologists physical status classification; WBC, white blood cell count; TSI, two-stage intervention; OR, odds ratio.

**Table 3:** Operative outcome analysis between one-stage intervention (OSI) group and two-stage intervention (TSI) group.

	Total (%/IQR)	OSI (%/IQR)	TSI (%/IQR)	*p*-Value
Post-Op days (d)	3.56 (3.396)	3.37 (2.934)	4.02 (4.121)	0.643
Op time (min)	124.39 (52.688)	118.01 (81.25–147.75)	125.76 (54.388)	0.869
Intra-operative blood transfusion (n)				1.000
Yes	2 (1.36)	1 (1.04)	1 (1.96)	
No	145 (98.64)	95 (98.96)	50 (98.04)	
Post-Op complication (n)				0.342
Yes	12 (8.16)	6 (6.25)	6 (11.76)	
No	135 (91.84)	90 (93.75)	45 (88.24)	
Conversion to open procedure (n)				0.236
Yes	7 (4.76)	3 (3.12)	4 (7.84)	
No	140 (95.24)	93 (96.88)	47 (92.16)	

IQR, interquartile range; Op, operation.

## Data Availability

Data available on request from the authors.

## References

[1] Miura F., Okamoto K., Takada T., Strasberg S.M., Asbun H.J., Pitt H.A., Gomi H., Solomkin J., Schlossberg D., Han H.-S. (2018). Tokyo Guidelines 2018: Initial management of acute biliary infection and flowchart for acute cholangitis. J. Hepato-Biliary-Pancreatic Sci..

[2] Okamoto K., Suzuki K., Takada T., Strasberg S.M., Asbun H.J., Endo I., Iwashita Y., Hibi T., Pitt H.A., Umezawa A. (2018). Tokyo Guidelines 2018: Flowchart for the management of acute cholecystitis. J. Hepato-Biliary-Pancreatic Sci..

[3] Ansaloni L., Pisano M., Coccolini F., Peitzmann A.B., Fingerhut A., Catena F., Agresta F., Allegri A., Bailey I., Balogh Z.J. (2016). 2016 WSES guidelines on acute calculous cholecystitis. World J. Emerg. Surg..

[4] JJoyce W.P., Keane R., Burke G.J., Daly M., Drumm J., Egan T.J., Delaney P.V. (1991). Identification of bile duct stones in patients undergoing laparoscopic cholecystectomy. Br. J. Surg..

[5] Manes G., Paspatis G., Aabakken L., Anderloni A., Arvanitakis M., Ah-Soune P., Barthet M., Domagk D., Dumonceau J.-M., Gigot J.-F. (2019). Endoscopic management of common bile duct stones: European Society of Gastrointestinal Endoscopy (ESGE) guideline. Endoscopy.

[6] Lee Y.T., Chan F.K., Leung W., Chan H.L., Wu J.C., Yung M.Y., Ng E.K., Lau J.Y., Sung J.J. (2008). Comparison of EUS and ERCP in the investigation with suspected biliary obstruction caused by choledocholithiasis: A randomized study. Gastrointest. Endosc..

[7] Lee J.K., Ryu J.K., Park J.K., Yoon W.J., Lee S.H., Lee K.H., Kim Y.T., Yoon Y.B. (2006). Risk factors of acute cholecystitis after endoscopic common bile duct stone removal. World J. Gastroenterol..

[8] Hung Y.-L., Chong S.-W., Cheng C.-T., Liao C.-H., Fu C.-Y., Hsieh C.-H., Yeh T.-S., Yeh C.-N., Jan Y.-Y., Wang S.-Y. (2020). Natural Course of Acute Cholecystitis in Patients Treated with Percutaneous Transhepatic Gallbladder Drainage without Elective Cholecystectomy. J. Gastrointest. Surg..

[9] Loozen C.S., Oor J.E., Van Ramshorst B., Van Santvoort H.C., Boerma D. (2017). Conservative treatment of acute cholecystitis: A systematic review and pooled analysis. Surg. Endosc..

[10] Pisano M., Allievi N., Gurusamy K., Borzellino G., Cimbanassi S., Boerna D., Coccolini F., Tufo A., Di Martino M., Leung J. (2020). 2020 World Society of Emergency Surgery updated guidelines for the diagnosis and treatment of acute calculus cholecystitis. World J. Emerg. Surg..

[11] da Costa D.W., A Bouwense S., Schepers N.J., Besselink M.G., van Santvoort H.C., van Brunschot S., Bakker O.J., Bollen T.L., Dejong C.H., van Goor H. (2015). Same-admission versus interval cholecystectomy for mild gallstone pancreatitis (PONCHO): A multicentre randomised controlled trial. Lancet.

[12] Abe T., Amano H., Hanada K., Bekki T., Minami T., Yonehara S., Noriyuki T., Nakahara M. (2019). Efficacy and safety of early cholecystectomy for comorbid acute cholecystitis and acute cholangitis: Retrospective cohort study. Ann. Med. Surg..

[13] Chen T.-S., Lin X.-H., Peng Y.-L., Luo J.-C., Chen Y.-T., Hou M.-C., Lee F.-Y. (2017). Cholecystectomy decreased the recurrent cholangitis after clearance of bile duct stones by ERCP in patients with gallstone-related cholangitis. J. Chin. Med. Assoc..

[14] Mador B.D., Nathens A.B., Xiong W., Panton O.N.M., Hameed S.M. (2017). Timing of cholecystectomy following endoscopic sphincterotomy: A population-based study. Surg. Endosc..

[15] El Nakeeb A., Ezzet H., Askar W., El Hanafy E., Hamdy E., Atef E., Youssef M., Talaat H., Hamed H., Abdallah T. (2016). Early Versus Late Cholecystectomy after Clearance of Common Bile Duct Stones by Endoscopic Retrograde Cholangiopancreatography: A Prospective Randomized Study. Surg. Laparosc. Endosc. Percutaneous Tech..

